# Intensive care admissions, experiences and outcomes for Indigenous children in Australia, Aotearoa (New Zealand), Canada and the USA: a scoping review protocol

**DOI:** 10.1136/bmjopen-2026-121647

**Published:** 2026-11-05

**Authors:** Roxanne Jones, Emily Colonna, Katherine Ann Thurber, Raymond Lovett

**Affiliations:** 1 Yardhura Walani, National Centre for Aboriginal and Torres Strait Islander Wellbeing Research, National Centre for Epidemiology and Population Health, Australian National University, Canberra, Australian Capital Territory, Australia

**Keywords:** Australian Aboriginal and Torres Strait Islander Peoples, INTENSIVE & CRITICAL CARE, Paediatric intensive & critical care, Paediatric intensive & critical care, Intensive Care Units

## Abstract

**Abstract:**

**Introduction:**

Critical illness during childhood and admissions to intensive care can have a profound impact on life trajectories, including long-term impacts on development, health, well-being, functional capacity and quality of life. For Indigenous peoples in settler-colonial nations, systemic factors, including racism and barriers to accessing healthcare, may shape interactions with intensive care. As survival rates following intensive care admission rise, there is a clear need for holistic information about the outcomes of critical illness for Indigenous children, beyond mortality-associated outcomes. This review will synthesise a diversity of evidence by extracting data on all characteristics of admissions to, experiences in, and outcomes during/following intensive care for Indigenous children under 16 years in settler-colonial nations: Canada, Australia, Aotearoa (New Zealand) and the USA (CANZUS nations).

**Methods and analysis:**

A search for peer-reviewed journal articles published in English or French in PubMed, ProQuest, Web of Science and SCOPUS databases from database inception to 1 September 2026 will be conducted. Grounded by an Indigenist standpoint and methodology, the Joanna Briggs Institute scoping review methodology will be followed and the Preferred Reporting Items for Systematic Reviews and Meta-Analyses extension for Scoping Reviews checklist will be reported against. Outcomes of interest include contextual factors (eg, remoteness), admission and clinical factors (eg, diagnoses), discharge outcomes (eg, disability), patient-reported experience and outcome measures and qualitative data on admission experiences. Articles selected for review will be grouped by study design, the Aboriginal and Torres Strait Islander Quality Appraisal Tool used to assess evidence quality and findings synthesised narratively. The research programme is guided by an Aboriginal and Torres Strait Islander reference group.

**Ethics and dissemination:**

Ethical approval has been granted by the Australian National University and the Australian Institute of Aboriginal and Torres Strait Islander Studies Human Research Ethics Committees. Results will be disseminated through peer-reviewed publication, presentations and community resource sheets.

**STRENGTHS AND LIMITATIONS OF THIS STUDY**

- Conducted from an Indigenist standpoint and with guidance from an Indigenous reference committee, cultural considerations are grounded in this review.
- Assessing articles against the Aboriginal and Torres Strait Islander Quality Appraisal Tool will identify if community and cultural knowledges were considered in the research, strengthening the contextual findings of this review.
- The scope and generalisability of the findings will be restricted by the exclusion of neonatal intensive care unit admissions and adolescents aged 16 years and over.
- A key limitation of the review is the focus on peer-reviewed evidence and the exclusion of a grey literature search, which may be a source of publication bias.

## Introduction

 The early years of life and into adolescence are a time of rapid development and are a key period for establishing a healthy lifecourse.^1 2^ Critical illness and injury during childhood and adolescence that requires admission to an intensive care unit (ICU) can have a profound impact on life trajectories.^3–6^ Impacts following critical illness and admissions to intensive care are multifaceted and far-reaching and may extend beyond the individual, to include siblings, caregivers and community.^6–9^ In Australia, Aboriginal and Torres Strait Islander children are admitted to ICU at a higher rate than non-Indigenous children (1.91 (95% CI 1.87 to 1.94) vs 1.60 (95% CI 1.57 to 1.64) per 1000 children per year) (Moynihan *et al*^10^). Aboriginal and Torres Strait Islander children also tend to present to ICU with a greater illness severity than non-Indigenous children^10 11^ and with differing patterns of illness and injury characteristics.^12–14^ For example, the proportion of admissions to ICU for severe infections (including sepsis) and trauma-related diagnoses has consistently been higher for Aboriginal and Torres Strait Islander children than non-Indigenous children over the last two decades.^10 12 15 16^ Between 2002 and 2013, the ICU admission rate for Aboriginal and Torres Strait Islander children presenting with an invasive infection was over three times the rate for Indigenous children compared with non-Indigenous children (47.6 vs 15.9 per 100 000 children per year).^16^ While there has been increased research investment in specific diagnostic fields such as sepsis, where documented inequities exist, a paucity of evidence remains that considers systemic factors that may influence these disparities and whether inequities exist across presentations and outcomes.

There is evidence to suggest that inequities in admission rates, illness and injury presentations, diagnostic and treatment characteristics, and outcomes of critical illness exist across the entire lifecourse for Indigenous peoples (compared with non-Indigenous peoples), including within neonatal,^17 18^ paediatric^10 16^ and adult^19–21^ ICUs and across settler-colonial nations.^7 22–25^ Historically, mortality has been the outcome of interest most researched in the intensive care literature; however, increasingly other outcome measures are being investigated as survival rates of critical illness and admissions to intensive care have increased over time.^6 26^ In recent years, mortality associated with paediatric intensive care admissions has been declining steadily in high income countries, while morbidity has increased.^3 11 26 27^ Long-term impacts on quality of life following critical illness and admission to paediatric intensive care for survivors can been seen across multiple health and well-being domains including social, emotional and psychological well-being; developmental, neurodevelopmental and educational outcomes and chronic disease and disability.^4 5 26 28^

Aboriginal and Torres Strait Islander people often have a much broader conceptualisation of health beyond an individualistic, biomedical, deficit-based model, to one that includes cultural determinants of health, and considers the collective health of family, community, kin and country as integral to the health of individuals.^29^ This holistic understanding of health is shared by many Indigenous populations around the world and is well documented across settler-colonial nations with similar healthcare systems such as Canada, Aotearoa (New Zealand) and the USA^30^ (CANZUS nations). Observed inequities in health outcomes between Indigenous and non-Indigenous peoples are not due to any underlying biological, racial or cultural difference; rather, they are reflective of historical and ongoing impacts of settler-colonialism and racism that generate and perpetuate health disparities.^23 31–34^ Indigenist research approaches represent research that is by, for, and with Indigenous peoples. Indigenist approaches are grounded in principles of resistance, political integrity and privileging Indigenous voices. Indigenist approaches bring to the fore the underlying drivers of inequity, such as settler-colonialism and racism.^35–37^

A systematic review of the intensive care literature identified 15 studies on the incidence and outcomes of critical illness for Indigenous adults (18 years and over) in Australia, Aotearoa and Canada, which found that Indigenous populations generally had higher rates of critical illness and ICU admission than non-Indigenous populations. The authors described the literature of Indigenous adults’ experiences with critical care as ‘poorly characterised’, that research rarely includes Indigenous perspectives or consultation and it is unclear whether communities have had any direct benefit from the research.^38^ This scoping review will focus on the paediatric population and aims to describe and synthesise the literature related to admissions to intensive care and outcomes of critical illness for Indigenous children in settler-colonial nations of Canada, Australia, Aotearoa and the USA.

A preliminary search of the Cochrane Database of Systematic Reviews and Joanna Briggs Institute (JBI) Evidence Synthesis databases did not identify any current or planned systematic or scoping reviews focusing on Indigenous children (under 16 years) admitted to intensive care, which this scoping review will investigate. This scoping review will extend on existing literature by describing and synthesising what is currently known of the experiences and outcomes of critical illness and admission to ICUs for Indigenous children, adopting a holistic frame to capture a broad range of information, beyond clinical outcomes. The review will appraise the methodological approaches of the literature with an Indigenist lens, summarising the involvement of Indigenous peoples in the project governance and leadership, development of the research priorities, interpretation and reporting of the findings. Scoping reviews use a systematic methodology to chart existing literature, synthesise key concepts, identify knowledge gaps, and summarise findings.^39 40^ A scoping review was chosen as the most appropriate method of evidence synthesis, as this research seeks to understand a diversity of evidence and the scoping review methodology allows for a broad approach, while maintaining methodological rigour.

### Objectives

The objective of this scoping review is to understand what is known about the experiences and outcomes of critical illness for Indigenous children admitted to paediatric ICUs in Australia, Aotearoa, Canada and the USA. This review also seeks to appraise the quality of the available evidence, from an Indigenous perspective, and highlight gaps for future research and investigation.

### Review questions

1. What evidence is available that describes the experiences and outcomes of critical illness and admissions to ICUs for Indigenous children in CANZUS nations?
2. What is the quality of the evidence identified in this review, from an Indigenous perspective, as assessed by the Aboriginal and Torres Strait Islander Quality Appraisal Tool?

### Research team positioning

RJ (Palawa) and RL (Ngiyampaa/Wongaibon) are Aboriginal researchers with backgrounds as Registered Nurses. RJ was previously a paediatric intensive care nurse which led to the initial conceptualisation of this project. KAT and EC (non-Indigenous) have experience in mixed-methods and strengths-based approaches within Aboriginal and Torres Strait Islander health research.

### Methodology

This review will be conducted by an Aboriginal-led research team and uses an Indigenist standpoint and methodology in addition to following the JBI scoping review guidelines, and will be reported in alignment with the Preferred Reporting Items for Systematic Reviews and Meta-Analyses extension for Scoping Reviews (PRISMA-ScR).^41^ Brodie *et al*^42^ note that integrating cultural ways (such as storytelling and sharing) when reviewing the literature systematically is not routine in health research, and argue that prioritising Indigenous ways of knowing, doing and being enhances scoping review methods.

Historically, Western research agendas have rarely considered the priorities of Indigenous peoples and perpetuated colonial power by using extractive methods and focusing on deficit discourses.^35 43^ Western research methodologies have actively excluded Indigenous voices and knowledge systems, while simultaneously conducting research ‘on’ but never ‘by’ Indigenous peoples, with little resultant change or improvement in health outcomes.^44^ Indigenist research epistemologies^35^ and Indigenous research methodologies^43–45^ challenge dominant Western approaches by centring and privileging Indigenous voices and knowledge systems.

Resistance in Indigenist research is founded in self-determination and the understanding of how colonialism has historically impacted, and continues to impact, the health and well-being of Indigenous Peoples, as seen across CANZUS nations.^31 33 46^ Political integrity in Indigenist research centres the research priorities of Indigenous Peoples, is undertaken with and by Indigenous Peoples, and crucially, it recognises that researchers are accountable to the communities in which they serve. The worldviews, lived experiences and aspirations of Indigenous Peoples are grounded in Indigenist approaches and power is shared by centring Indigenous Peoples as the primary population of interest in the research.^35^ This review seeks to enhance the existing literature by centring Indigenous knowledges and perspectives, such as the interconnectedness of kinship structures, connection to Country, family and community to the health and well-being of Indigenous children throughout the study development, analysis, reporting and translation of the findings.

This research programme will engage with an Indigenous reference group to guide the research and assist using a strengths-based approach, contextualising findings and assisting with presenting the results in a respectful and accessible way. The Thiitu Tharrmay (Ngiyampaa language, ‘to share/exchange knowledge’) reference group is a panel of Aboriginal and Torres Strait Islander experts that uphold Aboriginal and Torres Strait Islander ways of knowing, being and doing in research conducted at Yardhura Walani, the National Centre for Aboriginal and Torres Strait Islander Wellbeing Research. This mechanism encourages accountability and transparency to community through active partnerships and allows for an iterative process when planning research and describing and disseminating results. As an example, any results relating to end-of-life (Sorry Business) will be presented to the Thiitu Tharrmay reference group for input on how to most appropriately analyse and report on these outcomes.

## Methods and analysis

In alignment with best-practice guidelines for the conduct of scoping reviews, the proposed review will follow the JBI scoping review methodology. To assess the quality of the evidence identified in the review, the Aboriginal and Torres Strait Islander Quality Appraisal Tool will be used.^47^ Screening of articles for the review will commence immediately on acceptance of the protocol and the review is estimated to be complete by 31 December 2026.

### Inclusion and exclusion criteria

Peer-reviewed journal articles published between database inception and 1 September 2026 and published in English or French, will be eligible for inclusion. The target population of interest for this review are Indigenous children and adolescents aged under 16 years, that have been admitted to a paediatric ICU in a CANZUS nation. Critically ill neonates are a distinct population group with a distinct subspeciality that is beyond the scope of this review; therefore, articles that describe neonatal ICU admissions will be excluded. The inclusion criteria have been expanded below.

Population: Studies will be eligible for inclusion if the study population identifies as Indigenous (Aboriginal, Torres Strait Islander, Māori, First Nations, Inuit, Inuk, Métis, Cree, Alaska Native, Native Hawaiian or Native American). In alignment with the values of Indigenist research approaches, the population of interest in this review is Indigenous children and adolescents. Studies will be classified as a ‘primary’ source if the target population of the study was Indigenous children, or ‘incidental’ if Indigenous children were mentioned within a broader population but not an explicit focus of the research.
Concept: Studies that describe admissions to ICUs within settler-colonial CANZUS nations of Canada, Australia, Aotearoa and the USA are eligible for inclusion. Noting the limited availability of specialist paediatric intensive care services (geographical barriers, etc), studies will be deemed eligible for inclusion regardless of whether the participants were admitted to a mixed paediatric/adult ICU, mixed neonatal/paediatric ICU, general ICU or standalone paediatric ICU. Studies that describe only neonatal or adult intensive care admissions will be excluded from this review.
Context: The outcomes of interest include all characteristics of admissions to, experiences in and outcomes during/following intensive care for Indigenous children. For the purposes of this review, ‘critical care’ and ‘intensive care’ are used interchangeably.

### Information sources

This scoping review will consider peer-reviewed quantitative and qualitative study designs, in addition to reviews and protocols. Conference abstracts and theses will not be eligible for inclusion. We will conduct an electronic search of peer-reviewed articles published in PubMed, ProQuest, Web of Science and SCOPUS databases, using the Australian National University library subscriptions. A manual reference list search of articles included in this review will also be conducted. The search terms were refined, and the databases were selected following advice from an Australian National University librarian.

### Search strategy

The search strategy used in the proposed scoping review is detailed in the form of a flowchart at figure 1. A search will be conducted using the selected databases described above. Search results will be imported into Covidence and duplicates removed. Two reviewers will screen titles and abstracts and if deemed eligible for inclusion, will proceed to full-text screening. Reviewers will hand search reference lists of included articles for any further articles for inclusion in the review.

**Figure 1:**
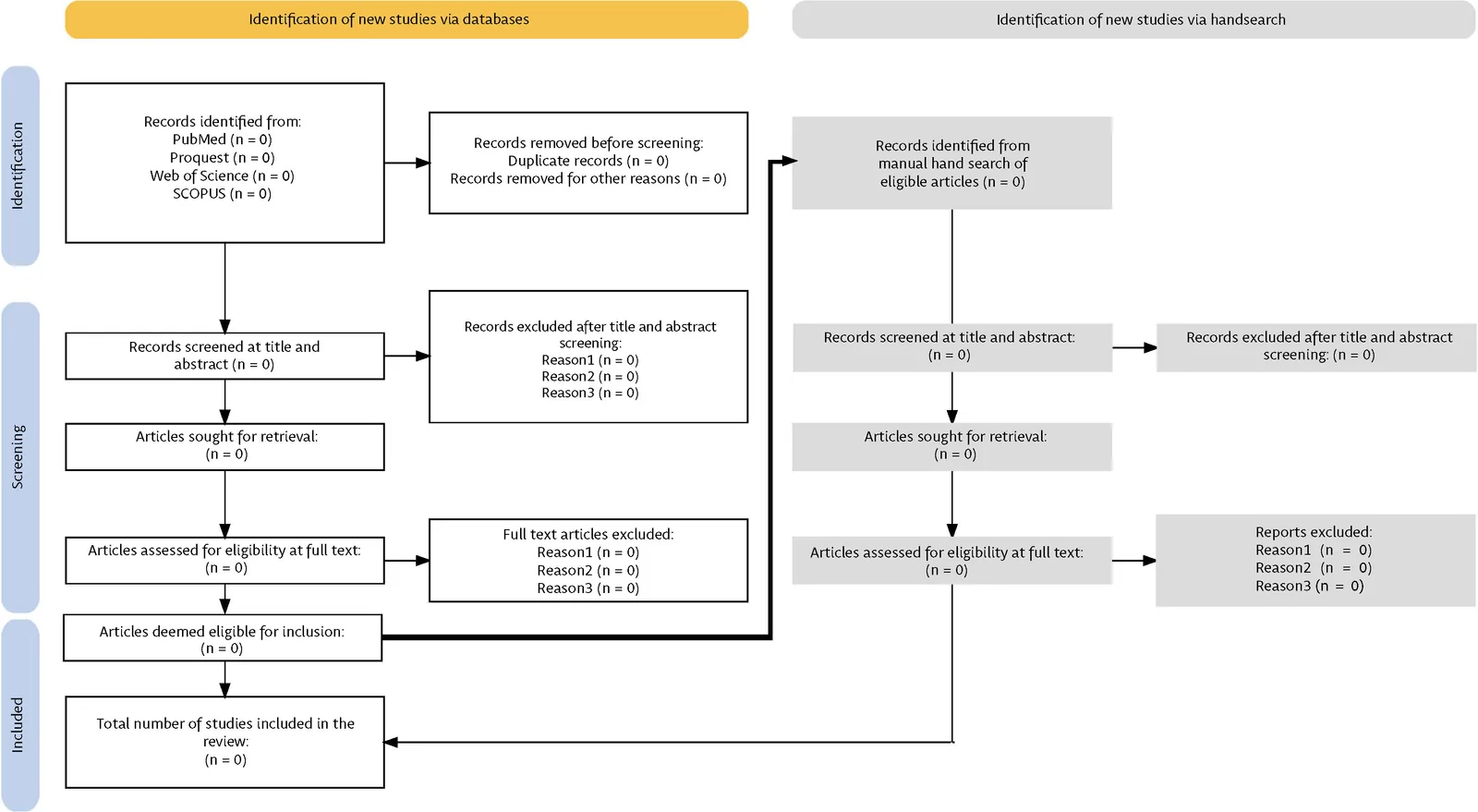
PRISMA flow chart. PRISMA, Preferred Reporting Items for Systematic Reviews and Meta-Analyses.

### Search terms

Preliminary search terms have been developed, and any necessary modifications will be reported on in the scoping review.

Population: (Aborig* OR “Torres Strait” OR “Torres Strait Islander” OR Indigenous OR “Indigenous peoples” OR “Australian Indigenous” OR “Indigenous Australia*” OR “Australian Aborig*” OR “Aboriginal Australian” OR “first nations” OR M?ori OR Inuit OR Inuk OR Metis OR Cree OR “Native American” OR “American Indian” OR “Native Alaskan” OR “Alaska Native” OR “Native Hawaiian” OR “Native Canadian”); AND
Concept: (paediatr* OR pediatr* OR child* OR preadolesc* OR adolesc* OR juvenile* OR teen* OR preteen* OR youth* OR “young people” OR kids OR boy* OR girl* OR prepubesc* OR pubescen* OR minor OR minors OR infant*); AND
Context: (“intensive care” OR “critical care”).

### Source of evidence selection

All studies identified during the search will be imported into Covidence. Covidence functionality allows for duplicates to be removed and the effective screening and organising of articles with more than one reviewer. Titles and abstracts will be screened against the inclusion criteria and those identified as potentially relevant will have the full-text retrieved. These selected articles will have the full-text reviewed against the inclusion criteria and checked by the first author and a reviewer. Reasons for exclusion of articles that do not meet the inclusion criteria will be recorded and reported on in the scoping review. Any disagreements that arise during the review and selection process will be discussed with the author group, or with the assistance of an additional reviewer. The search results and studies for inclusion will be reported on in full in the final scoping review.

### Data items and extraction

The data extraction process will seek to gather descriptive information on each article such as author, date and country. It will also gather additional information relative to the review including: study population (age range, sample size, Indigenous group), aim, data source, ICU type, study design and methods, contextual factors (such as consideration of impact of colonisation), key findings and recommendations. Specific outcomes of interest that will be extracted (if present) include: length of stay, study focus area (such as sepsis), pre-ICU admission characteristics, ICU admission characteristics (such as reason for admission), ICU outcome measures, chronic comorbidities, long-term postdischarge outcomes, patient-reported outcome measures and patient-reported experience measures, and mortality. Qualitative data on the experiences of admissions to ICU will be summarised and charted if present. Data extraction will occur in Covidence by the first author and summarised in table 1.

**Table 1:** Data extraction priorities

Data extraction priorities (example data to be extracted in non-bold)	Articles for inclusion (N=x)	
Review number	#001	#002
Author and year		
Title		
Study population Age range, sample size, Indigenous group		
Aim		
Country Australia, Canada, Aotearoa, USA		
Intensive care unit type Paediatric intensive care unit, general intensive care unit		
Data source Registries, government statistical agencies		
Study design and methods Qualitative, quantitative, case-report, clinical trial		
Contextual factors considered Social and cultural determinants of health; racism and colonisation		
Study key focus area Sepsis, trauma, infectious disease, cancer, surgical		
Clinical outcome measures Length of stay, postintensive care syndrome,		
Chronic comorbidities Life-limiting conditions, medical complexity, chronic disease		
Patient-reported experience measures Patient-reported or caregiver-proxy measures		
Patient-reported outcome measures Health-related quality of life		
Key findings and recommendations		

To assess the quality of the selected evidence, articles will be assessed in Covidence by two reviewers and data will be exported into an excel file. Harfield *et al*^47^ developed and validated the quality appraisal tool in response to a need to contextually appraise research from an Aboriginal and Torres Strait Islander perspective. Similar to Bowker *et al*^38^ and as discussed by Harfield *et al*,^48^ we have slightly modified the language for the purposes of this review to reflect all Indigenous peoples, and as such will be referred to herein as the Indigenous Quality Appraisal Tool (IQAT). Where the tool describes ‘Aboriginal and Torres Strait Islander people’, these have been replaced with ‘Indigenous’ (summarised in table 2).

**Table 2:** Indigenous Quality Appraisal Tool assessment of articles included in the scoping review

Indigenous Quality Appraisal Tool scoring* chart	Articles for inclusion (N=x)	
Review number, author, year	#001	#002:
Q1: Did the research respond to a need or priority determined by the community?	Y (1)	
Q2: Was community consultation and engagement appropriately inclusive?	Y (1)	
Q3: Did the research have Indigenous research leadership?	Y (1)	
Q4: Did the research have Indigenous governance?	P (0.5)	
Q5: Were local community protocols respected and followed?	P (0.5)	
Q6: Did the researchers negotiate agreements in regard to rights of access to Indigenous peoples’ existing intellectual and cultural property?	P (0.5)	
Q7: Did the researchers negotiate agreements to protect Indigenous peoples’ ownership of intellectual and cultural property created through the research?	N (0)	
Q8: Did Indigenous peoples and communities have control over the collection and management of research materials?	N (0)	
Q9: Was the research guided by an Indigenous research paradigm?	N (0)	
Q10: Does the research take a strength-based approach, acknowledging and moving beyond practices that have harmed Indigenous peoples’ in the past?	U (0)	
Q11: Did the researchers plan and translate the findings into sustainable changes in policy and/or practice?	U (0)	
Q12: Did the research benefit the participants and Indigenous communities?	U (0)	
Q13: Did the research demonstrate capacity strengthening for Indigenous individuals?	P (0.5)	
Q14: Did everyone involved in the research have opportunities to learn from each other?	Y (1)	
Total score (up to 14 points)	6	

* Yes=Y (1 point); Partially=P (0.5 points); No=N (0 points) Unsure=U (0 points).

Two reviewers will appraise each study in Covidence. Each response will be allocated points and colour coded for analysis: yes (green) 1 point, partially (yellow) 0.5 point, unclear (grey) 0 point, no (red) 0 point. Any disagreements that arise during the appraisal will be discussed with the author group, or with the assistance of an additional reviewer. Similar to Colonna *et al*^49^ an implementation guide (online supplemental file B) will accompany the IQAT and support consistency of interpretation by reviewers.

### Evidence synthesis

Data on study characteristics will be presented (table 1), summarised and grouped by study design (for example, quantitative or qualitative). A narrative summary will contextualise the findings, in alignment with the scoping review questions and objectives. If present, qualitative data will supplement the narrative review of the quantitative data. Evidence will be presented by study and by IQAT question (tables 2 and 3). Results and analysis will be presented to Thiitu Tharrmay for input to contextualise the findings and that the reporting of results reflects community expectations.

**Table 3:** Distribution of Indigenous Quality Appraisal Tool scoring across included articles, by item (N=(total number of reviews))

The Indigenous Quality Appraisal Tool		Response options			
		Yes n (%)	Partially n (%)	No n (%)	Unclear n (%)
Q1	Did the research respond to a need or priority determined by the community?				
Q2	Was community consultation and engagement appropriately inclusive?				
Q3	Did the research have Indigenous research leadership?				
Q4	Did the research have Indigenous governance?				
Q5	Were local community protocols respected and followed?				
Q6	Did the researchers negotiate agreements in regard to rights of access to Indigenous peoples’ existing intellectual and cultural property?				
Q7	Did the researchers negotiate agreements to protect Indigenous peoples’ ownership of intellectual and cultural property created through the research?				
Q8	Did Indigenous peoples and communities have control over the collection and management of research materials?				
Q9	Was the research guided by an Indigenous research paradigm?				
Q10	Does the research take a strength-based approach, acknowledging and moving beyond practices that have harmed Indigenous peoples in the past?				
Q11	Did the researchers plan and translate the findings into sustainable changes in policy and/or practice?				
Q12	Did the research benefit the participants and Indigenous communities?				
Q13	Did the research demonstrate capacity strengthening for Indigenous individuals?				
Q14	Did everyone involved in the research have opportunities to learn from each other?				

### Limitations

The completeness of Indigenous identity variables in routine healthcare and administrative datasets, and in research reporting, has been historically inconsistent due to systemic and institutional barriers and can vary across states and nations. Misclassification of Indigenous identification in datasets may result in underestimation of Indigenous admissions and outcomes which will be a noted limitation in the analysis. Our search will be restricted to peer-reviewed articles published in indexed databases to ensure rigour and support feasibility. The exclusion of grey literature may result in fewer articles being eligible for inclusion.

### Patient and public involvement

This protocol and scoping review do not include direct patient or public contact, or primary data collection. An Aboriginal and Torres Strait Islander reference group of community and experts with lived experience has been engaged throughout this research programme. Further details are provided in the Methodology section.

## Ethics and dissemination

This article reports a protocol for a scoping review of existing published literature and no direct contact or primary data collection from human participants is conducted. However, the review does conduct a secondary analysis of human data; therefore, the broader research programme has been approved by two national Human Research and Ethics Committees (HREC) from the Australian Institute of Aboriginal and Torres Strait Islander Studies (AIATSIS) (EO291-20210830) and the Australian National University (ANU) (2023/436). This article is not linked to a clinical trial. Results will be disseminated through peer-reviewed publication, institutional and national seminars and presentations, alongside a community resource with input from the Thiitu Tharrmay reference group.

## Supplementary material

10.1136/bmjopen-2026-121647 online supplemental file 1
