# Supplementary material for: Intensive care admissions, experiences and outcomes for Indigenous children in Australia, Aotearoa (New Zealand), Canada and the USA: a scoping review protocol
Source: BMJ Open. 2026 Nov 5;16(11):e121647. doi: 10.1136/bmjopen-2026-121647 (PMC13756485; doi:10.1136/bmjopen-2026-121647)
Supplement: Supplementary file 1 [file bmjopen-2026-121647.reviewer_comments.pdf]

PEER REVIEW HISTORY

BMJ Open publishes all reviews undertaken for accepted manuscripts. Reviewers are asked to complete a checklist review form and are provided with free text boxes to elaborate on their assessment. These free text comments are reproduced below.

ARTICLE DETAILS

Title (Provisional)

Intensive care admissions, experiences and outcomes for Indigenous children in Australia, Aotearoa (New Zealand), Canada and the United States: a scoping review protocol

Authors

Jones, Roxanne; Colonna, Emily; Thurber, Katherine Ann; Lovett, Raymond

---

VERSION 1 - REVIEW

**Reviewer**	**1**
**Name**	**Ante-Ardila, Natalia**
**Affiliation**	**Hospital Universitario de la Fundación Santa Fe de Bogotá, Pediatrics**
**Date**	**13-Jul-2026**
**COI**	**None**

I consider this a good protocol, given the limited information available in the literature on this specific population.

I recommend making the following adjustments for publication:

Abstract: It is recommended to explicitly mention in the abstract that the protocol follows the PRISMA-ScR guidelines and the Joanna Briggs Institute (JBI) methodology.

Introduction:

1. Define the acronym CANZUS the first time it is mentioned.

Inclusion criteria:

2. How to control for information bias, given that Indigenous ethnicity is often underreported in medical records, which can affect the available literature?

Methodology:

3. Scoping reviews, by definition, seek to map all available evidence. In topics concerning the health of marginalized or Indigenous populations, a large portion of valuable information (epidemiological reports, care protocols, hospital metrics) is found in "grey literature"

(government reports, Indigenous health organization documents). It is strongly recommended to include a gray literature search strategy, or alternatively, to justify in the "Limitations" section why it was excluded.

4. By including Canada (a CANZUS nation), restricting the language solely to English could exclude relevant clinical literature from Francophone provinces, which have significant populations (e.g., Indigenous First Nations, Inuit, Métis). It is suggested to justify this language exclusion.

Data extraction:

5. In Table 1, within the "Clinical focus area", it would be worthwhile to include whether the study mentions previous pediatric comorbidities (e.g., asthma, malnutrition, early endocrine or metabolic pathologies), as these directly influence the severity of ICU admission and the clinical recovery trajectory.

I congratulate you on the protocol, I feel that it has several strengths:

- Clear and Relevant Focus: The research objective successfully shifts the focus from mere mortality rates to a comprehensive evaluation of morbidity, long-term development impacts, and patient experiences for Indigenous children in intensive care units.

- Strong Indigenous Methodology: The study design is deeply rooted in an Indigenous approach, notably governed by a dedicated reference group (Thiitu Tharrmay), ensuring exceptional cultural validity and relevance.

- Innovative Quality Assessment: The implementation and linguistic adaptation of the Indigenous Quality Appraisal Tool (IQAT) provide a highly reproducible, ethical, and rigorous framework for evaluating primary studies.

- Comprehensive Data Extraction: The planned extraction matrix thoroughly covers crucial variables, including pre-admission characteristics, clinical diagnoses, discharge outcomes, and patient-reported measures.

- Ethical Rigor: By acknowledging the historical and ongoing impacts of settler-colonialism rather than attributing health disparities to biological differences, the protocol demonstrates a profound ethical respect for the studied population.

Congratulations, I'd be delighted to see the suggested corrections, their publication, and later, the results of the review.

VERSION 1 - AUTHOR RESPONSE

Reviewer 1 Comments

R1: It is recommended to explicitly mention in the abstract that the protocol follows the PRISMA-ScR guidelines and the Joanna Briggs Institute (JBI) methodology

We have restructured and reworded the abstract (as above) and specified that we will be following the PRISMA-ScR guidelines and JBI methodology. It is specifically mentioned at lines 51-53.

Grounded by an Indigenist standpoint and methodology, the Joanna Briggs Institute scoping review methodology will be followed and the Preferred Reporting Items for Systematic and Meta-Analyses extension for Scoping Reviews checklist will be reported against.

R2: Define the acronym CANZUS the first time it is mentioned.

We have altered the title of the manuscript to remove the CANZUS acronym and have ensured the acronym is defined at the first mention in-text and within each section (at lines 2, 47 and 116).

R3: Inclusion criteria: How to control for information bias, given that Indigenous ethnicity is often underreported in medical records, which can affect the available literature?

We have noted the underreporting of Indigenous identity variables within health records at the new limitations section at lines 326-332 and expanded our response below under R4.

R4: Scoping reviews, by definition, seek to map all available evidence. In topics concerning the health of marginalized or Indigenous populations, a large portion of valuable information (epidemiological reports, care protocols, hospital metrics) is found in "grey literature" (government reports, Indigenous health organization documents). It is strongly recommended to include a gray literature search strategy, or alternatively, to justify in the "Limitations" section why it was excluded.

Thank you for identifying this limitation and for the recommendations. The team understands the concern and has discussed this in depth and drafted a new limitations section at the end of the manuscript in response. This review forms part of a PhD project and therefore has some resource constraints (notably time and personnel for full-text reviewing) that impact the feasibility of conducting a grey literature search as part of this review.

At lines 326-332, this has now been articulated in the manuscript and will be emphasised in the review: *The completeness of Indigenous identity variables in routine healthcare and administrative datasets, and in research reporting, has been historically inconsistent due to systemic and institutional barriers and can vary across states and nations. Misclassification of Indigenous identification in datasets may result in underestimation of Indigenous admissions and outcomes which will be a noted limitation in the analysis. Our search will be restricted to peer-reviewed articles published in indexed databases to ensure rigour and support feasibility. The exclusion of grey literature may result in fewer articles being eligible for inclusion.*

R5: By including Canada (a CANZUS nation), restricting the language solely to English could exclude relevant clinical literature from Francophone provinces, which have significant populations (e.g., Indigenous First Nations, Inuit, Métis). It is suggested to justify this language exclusion.

Thank you for the opportunity to consider this important issue. We have made changes within the manuscript to clarify that studies published in French will also be eligible for inclusion.

Line 50: *An electronic search for peer-reviewed journal articles published in English or French in PubMed, ProQuest, Web of Science, and SCOPUS databases prior to 01-September-2026 will be conducted.*

Line 215: *Peer-reviewed journal articles published before 1 September 2026, and published in English or French, will be eligible for inclusion.*

R6: In Table 1, within the "Clinical focus area", it would be worthwhile to include whether the study mentions previous pediatric comorbidities (e.g., asthma, malnutrition, early endocrine or metabolic pathologies), as these directly influence the severity of ICU admission and the clinical recovery trajectory.

Thank you for identifying that the in-text outcomes of interest were not all accurately represented in Table 1. We have added a ‘Chronic comorbidities’ section to Table 1 at line 293 and added additional clarifying statements.
