# Supplementary material for: Intensive care admissions, experiences and outcomes for Indigenous children in Australia, Aotearoa (New Zealand), Canada and the USA: a scoping review protocol
Source: BMJ Open. 2026 Nov 5;16(11):e121647. doi: 10.1136/bmjopen-2026-121647 (PMC13756485; doi:10.1136/bmjopen-2026-121647)
Supplement: Supplementary file 2 [file bmjopen-16-11-s001.docx]

**Supplementary material B:** **Implementation guide for interpreting the Indigenous Quality Appraisal Tool**

Background summary

Harfield et al. (2020) developed and validated the quality appraisal tool in response to a need to contextually appraise research from an Indigenous perspective. We have slightly modified the language in the IQAT for the purposes of this review to reflect all Indigenous Peoples, and as such will be referred to herein as the Indigenous Quality Appraisal Tool (IQAT). Where the tool describes ‘Aboriginal and Torres Strait Islander people’, these have been replaced with ‘Indigenous’.

The authors identified a need for clarity between reviewers on how to interpret and report on the IQAT with consistency between reviewers. Harfield et al. (2020) describes the response options and provides brief clarifying statements to assist reviewers:

- *Yes: provide clear written evidence in favour of the question*
- *Partial: provide some evidence or brief evidence in favour of the question*
- *Unclear: may have actioned the question item but did not explicitly state full or partial evidence*
- *No: reported evidence not in favour of the question*

Implementation guide

Two reviewers will appraise each study in Covidence utilising the IQAT response options: yes (Y), partially (P), no (N), unclear (U) and, extract, summarise and score. Any disagreements that arise during the appraisal will be discussed with the whole author group, or with the assistance of an additional reviewer. Any necessary modifications to the implementation guide will be reported on in the scoping review.

Each study will be appraised and interpreted with the assistance of the IQAT implementation matrix (Table 5), with each response allocated points: yes – 1 point; partially – 0.5 point; unclear – 0 points; no – 0 points. The sum total score will be presented for each included article.

Table 5: IQAT implementation matrix

| The Indigenous Quality Appraisal Tool | | Response options and interpretation guide | |
| --- | --- | --- | --- |
| Q1 | Did the research respond to a need or priority determined by the community? | Yes | Clear evidence of: community identified priority; community approaching researcher; emerging issues identified by community etc. |
|  |  | Partially | Some evidence of the above |
|  |  | No | Evidence to the contrary; priorities determined by non-Indigenous stakeholders |
|  |  | Unclear | Not reported |
| Q2 | Was community consultation and engagement appropriately inclusive? | Yes | Clear evidence of: consultation prior to research commencing; diverse consultation with community groups; multiple episodes of engagement; a change in question, method or analysis as a result of consultation with community |
|  |  | Partially | Singular episode of engagement |
|  |  | No | No evidence of consultation or engagement |
|  |  | Unclear | Some minor evidence of attempts to consult or engage but reporting is unclear |
| Q3 | Did the research have Indigenous research leadership? | Yes | First or senior author is Indigenous |
|  |  | Partially | At least one Indigenous co-author and evidence of their input in the design, analysis and interpretation of the data |
|  |  | No | No evidence of Indigenous authorship |
|  |  | Unclear | Some evidence of Indigenous leadership in project but not explicitly stated in authorship |
| Q4 | Did the research have Indigenous governance? | Yes | Clear evidence that a group of Indigenous peoples had decision making authority throughout the project; Indigenous peoples had control throughout all research phases; reported evidence of how the governance structure informed/altered/monitored the research |
|  |  | Partially | Some evidence of Indigenous governance but lacks clarity on how the governance has been applied to the research |
|  |  | No | No evidence of Indigenous governance; evidence of non-Indigenous governance |
|  |  | Unclear | Evidence of governance but unclear if that the governance mechanism includes Indigenous people |
| Q5 | Were local community protocols respected and followed? | Yes | Evidence that community protocols were identified and followed; evidence of a change in research approach following local community input; interpreters available if required; evidence of a change in timing/resources/engagement that is adaptive to community needs (for example: delaying data collection in respect of Sorry Business) |
|  |  | Partially | Some evidence reported |
|  |  | No | Evidence to the contrary |
|  |  | Unclear | Not reported |
| Q6 | Did the researchers negotiate agreements in regard to rights of access to Indigenous peoples’ existing intellectual and cultural property? | Yes | Clear evidence of a formal agreement negotiated between the researchers and community/individuals with existing Indigenous intellectual and cultural property |
|  |  | Partially | Some evidence of negotiation but no formal agreement |
|  |  | No | Not reported; evidence to the contrary |
|  |  | Unclear | Intellectual and cultural property rights have been briefly mentioned but no explicit details |
| Q7 | Did the researchers negotiate agreements to protect Indigenous peoples’ ownership of intellectual and cultural property created through the research? | Yes | Clear evidence of a formal agreement negotiated between the researchers and community/individuals for Indigenous intellectual and cultural property created from the research; clear declaration of ownership of intellectual and cultural property created from the research |
|  |  | Partially | Some evidence of negotiation but no formal agreement |
|  |  | No | Not reported; evidence to the contrary |
|  |  | Unclear | Intellectual and cultural property has been mentioned but no explicit details |
| Q8 | Did Indigenous peoples and communities have control over the collection and management of research materials? | Yes | Evidence that Indigenous peoples and communities have meaningful control in the development and application of appropriate data collection and management processes; participants have control over how their data is used and stored; processes clearly articulated in participant consent forms; ……. |
|  |  | Partially | Some evidence but lacks explicit detail |
|  |  | No | Evidence to the contrary |
|  |  | Unclear | Not reported |
| Q9 | Was the research guided by an Indigenous research paradigm? | Yes | An Indigenous research paradigm/methodology was used, with a clear description of how it reflects Aboriginal and Torres Strait Islander ways of knowing, being and doing; Indigenous people and communities informed the entire research lifecycle, including engagement, conceptualisation, implementation, interpretation and dissemination of research findings; a description of researcher engagement, prior to and during the research, to understand the perspectives of Indigenous people and communities, with a description of how this knowledge has informed the research |
|  |  | Partially | Some evidence that an Indigenous research paradigm/methodology was utilised at some stage of the research but not through the entirety of the research lifecycle; some evidence of attempts to incorporate Indigenous research paradigms/methodologies |
|  |  | No | Evidence to the contrary; Indigenous research paradigms or methodologies not mentioned or reflected upon |
|  |  | Unclear | Minor evidence that Indigenous research worldviews, paradigms and methodologies have been considered but unclear how they influenced the research approach and analysis |
| Q10 | Does the research take a strength-based approach, acknowledging and moving beyond practices that have harmed Indigenous peoples in the past? | Yes | Evidence of acknowledging past harms; evidence of a strengths-based approach being utilised throughout the design, implementation and dissemination of findings |
|  |  | Partially | Some evidence of acknowledging prior harms; some evidence of utilising a strengths-based approach |
|  |  | No | Evidence to the contrary; deficits-based approach or language |
|  |  | Unclear | Mixed or inconsistent evidence |
| Q11 | Did the researchers plan and translate the findings into sustainable changes in policy and/or practice? | Yes | Evidence of implementation of a knowledge translation plan; knowledge translation plan ensures Indigenous people and communities receive the results; Indigenous people and communities are involved in translating the research into sustainable policy and practice change; evidence of a change in policy or practice as a result of the research |
|  |  | Partially | Some minor evidence of a knowledge translation plan; some minor evidence of dissemination; some evidence of consideration of policy/practice implications |
|  |  | No | Evidence to the contrary |
|  |  | Unclear | Not reported |
| Q12 | Did the research benefit the participants and Indigenous communities? | Yes | Benefit to participants and Indigenous communities considered and stated; the research addressed community needs; evidence of an ongoing resource for Indigenous communities as a product of the research; evidence of investment in funding or programs as a direct result of the research; evidence of delivery of a service to Indigenous communities |
|  |  | Partially | Some minor evidence of benefit to participants and Indigenous communities but lacks detail |
|  |  | No | Evidence to the contrary; benefits for non-Indigenous stakeholders only |
|  |  | Unclear | Not reported |
| Q13 | Did the research demonstrate capacity strengthening for Indigenous individuals? | Yes | Evidence of employment, training or capacity building (formal or informal) for Indigenous people during and after the research |
|  |  | Partially | Some evidence of the above but lacks specific detail |
|  |  | No | Evidence to the contrary |
|  |  | Unclear | Not reported |
| Q14 | Did everyone involved in the research have opportunities to learn from each other? | Yes | Evidence of meaningful partnership between Indigenous and non-Indigenous researchers; evidence of meaningful partnership between Indigenous communities and researchers; evidence of change to the research process as a result of engagement with Indigenous communities; evidence of researchers planning to or having delivered the results back to participants and Indigenous communities in an accessible and useful manner |
|  |  | Partially | Some evidence of the above but not detailed or ad hoc |
|  |  | No | Evidence to the contrary |
|  |  | Unclear | Not reported |
